# Challenges for Developing Palliative Care Services in Resource- Limited Settings of Kazakhstan

**DOI:** 10.3389/phrs.2023.1605672

**Published:** 2023-08-18

**Authors:** Islam Salikhanov, Stephen R. Connor, Gulnara Kunirova, Fatima Khashagulgova, Gulzhaina Nazarova, Byron Lawrence Crape, Maria C. Katapodi

**Affiliations:** ^1^ Department of Clinical Research, University of Basel, Basel, Switzerland; ^2^ Worldwide Hospice Palliative Care Alliance, Fairfax, VA, United States; ^3^ Kazakhstan Palliative Care Association, Almaty, Kazakhstan; ^4^ National Research Cardiac Surgery Center, Astana, Kazakhstan; ^5^ School of Medicine, Nazarbayev University, Nur-Sultan, Kazakhstan

**Keywords:** challenges, health policy, Kazakhstan, low-middle income countries, palliative care

## Abstract

**Background:** Approximately 40 million people in need of palliative care worldwide, while 80% of them live in low- and middle-income countries. Kazakhstan, a low-to middle-income country with a reforming healthcare system, is committed to improving quality and accessibility of care for its 100,000 terminal patients in need of palliative care.

**Policy Options and Recommendations:** To join the group of countries where palliative care is available, accessible, and affordable, Kazakhstan must integrate palliative services into the mainstream healthcare system at all levels, from primary healthcare to hospices, and from major cities to remote villages. Based on the evidence thoroughly collected directly from the Ministry of Health, authors propose a feasible set of recommendations regarding palliative policy, pain relief, infrastructure, workforce, and education, which could be implemented in LMICs beyond Kazakhstan.

**Conclusion:** This study presents an analysis of challenges, recent developments, and needs of palliative care in Kazakhstan, including funding, policy, workforce, education, and infrastructure, providing an evidence base and recommendations for future development of palliative care in Kazakhstan and in other LMICs.

## Background

According to WHO, of the 40 million people who require palliative care worldwide, only 12% receive it, most of whom live in high-income countries [1]. With a population of approximately 19 million living in an area of 2.7 million ${km}^{2}$ , Kazakhstan has one of the lowest population densities worldwide, with only seven people per ${km}^{2}$ [2]. According to the 2nd edition of the Global Atlas of Palliative Care, currently 107,430 patients in Kazakhstan require palliative care, including 4,900 children [3]. In 2021, there were about 35,000 newly diagnosed cancer cases and approximately 190,000 people living with cancer in Kazakhstan, while more than 14,000 people died from the disease [4]. In addition, Kazakhstan has a total incidence of 60 tuberculosis cases per 100,000, where the registration of 50 cases per 100,000 is considered an epidemic [5]. This places Kazakhstan among the top 30 countries worldwide with the highest burden of multidrug-resistant tuberculosis [5]. The dispersed population poses serious challenges for accessing health services, especially for half the population in Kazakhstan who live in rural areas [6]. Despite being a relatively young nation, Kazakhstan needs to anticipate these trends, and the consequences and problems caused by the rise of chronic non-communicable diseases such as cancer, diabetes, and cardiovascular disease [7, 8]. As the demographic shift continues in LMICs, aging and palliative care needs will become increasingly linked [9]. The purpose of this policy brief is to conduct a detailed assessment and discuss the challenges of palliative care services in Kazakhstan, thereby informing researchers, LMICs policymakers, and other essential palliative care measurement initiatives, such as the Global Atlas of Palliative Care, the Lancet Commission on Palliative Care and Pain Relief, and Quality of Death and Dying Index [1, 3, 10].

## Evidence

### Global Perspective on Palliative Care in Kazakhstan

Target 3.8 of the Sustainable Development Goals adopted by the United Nations in 2015 indicates the necessity to achieve universal health coverage (UHC), including access to essential healthcare services and medicines, and financial risk protection for all by the year 2030 [11]. According to the description of services under UHC no nation can achieve true universal health coverage without including palliative care [9]. The 2015 Quality of Death Index, which evaluates quality of palliative care worldwide, ranked Kazakhstan 50th out of 80 countries assessed [10]. Palliative care in Kazakhstan has a government-led development strategy, which represents a statement of intent but needs a clearer vision and better mechanisms in place [2]. One of the reasons for this low ranking is the rampant incidence of unrelieved pain among most patients (8 points on a 10-point scale), and the low prioritization of developing palliative care services [10]. According to the Global Atlas of Palliative Care, rankings of palliative care in Kazakhstan have improved from the level which characterizes countries with general provision of palliative care, to the level which defines palliative systems at preliminary stage of integration to the healthcare system [3]. Kazakhstan is currently classified as having a diverse workforce of palliative care providers and types of services, healthcare professionals and local communities are aware of palliative care; and a palliative care strategy is being implemented and is regularly evaluated [3].

### Policy

In 2020, the Kazakh government introduced a new National Palliative Care Standard, which now assists the integration of palliative care into the primary healthcare system, ensuring continuity of care. According to the Standard, multidisciplinary palliative teams should be created in all major medical centers, general hospitals, and specialized clinics of the republican level. As part of the National Cancer Control Plan, terminal cancer patients across the country have access to mobile teams that provide in-home palliative care [12]. Moreover, 17 essential medicines, including 3 opioids, have been included into the free palliative package [13, 14]. As a United Nations member state working with WHO, Kazakhstan has committed to integrating palliative care into the healthcare system by signing the wide range of international initiatives in the field of health and human rights [15, 16]. The Kazakhstan Palliative Care Association (KPCA) has a leading role in palliative care advocacy, education, training, and government outreach, raising awareness and stimulating volunteerism. Given the absence of a palliative care registry, KAPC conducts continuous needs assessments, collects data on infrastructure, participates in the international agenda for palliative care development, and cooperates with the Ministry of Health. In 2018, the adoption of the first roadmap of palliative care development significantly improved care quality, knowledge, coverage, and awareness. The Ministry of Health updated the roadmap in 2022, with goals to enhance legal and regulatory frameworks, expand medication and equipment coverage, improve opioid accessibility, and establish better monitoring indicators.

### Infrastructure

**FIGURE 1:**
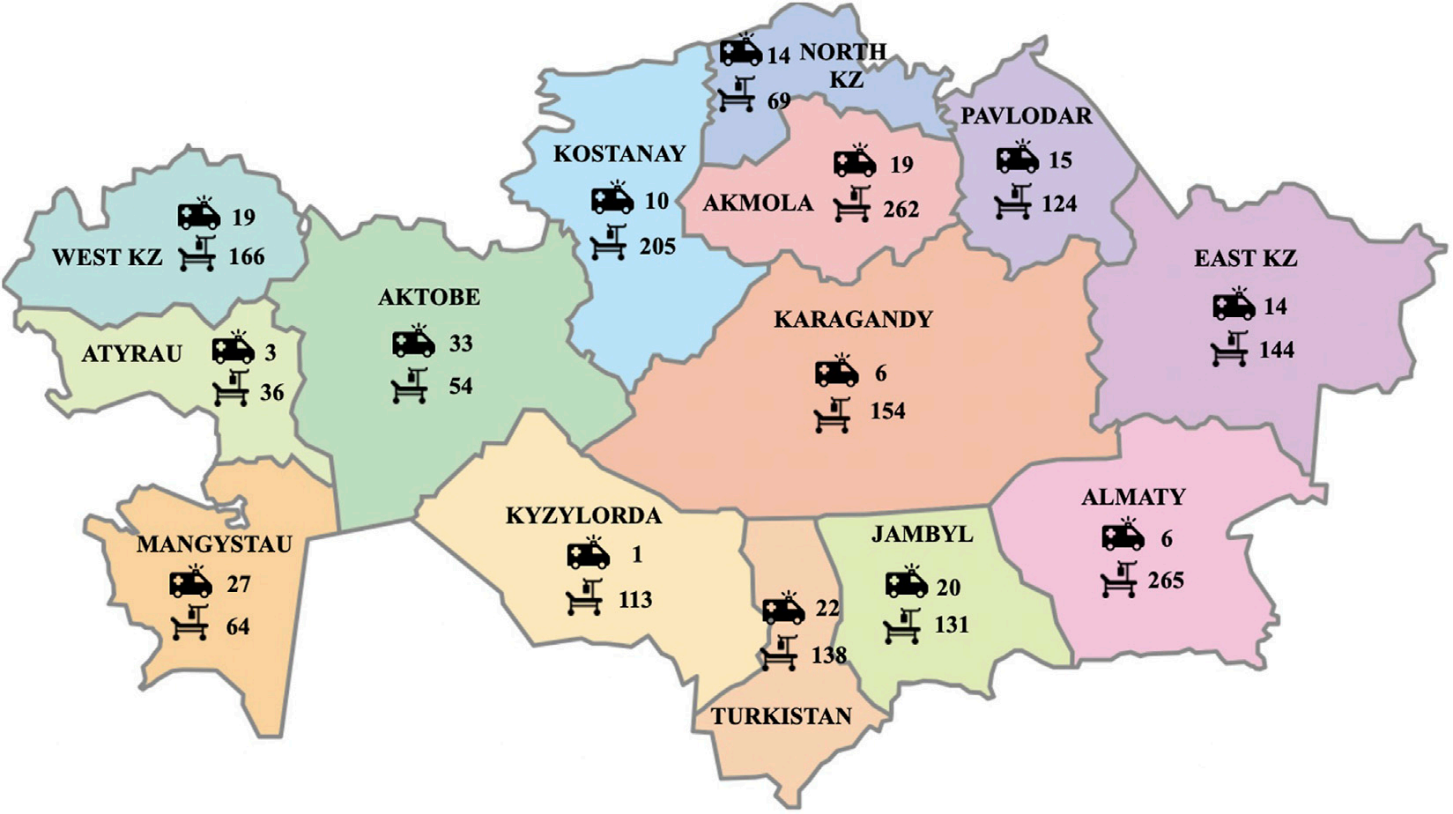
Distribution of palliative mobile teams and in-patient palliative beds across 14 regions of Kazakhstan (National Research Center for Health Development) (Kazakhstan, 2020–2023).

Currently, in-patient palliative care in Kazakhstan is provided mainly in stand-alone hospices and palliative care units of specialized cancer centers or general hospitals (see Figure 1). There are currently 12 hospices located in largest cities while palliative care units have been established as an integral part of cancer centers, distributed across all regions of the country under the “Comprehensive Cancer Control Plan for 2018–2022” [12]. In 2018, under this plan, mobile teams were introduced for in-home palliative care for cancer patients. The only state financed specialized pediatric palliative unit is located in Shymkent with a focus on children with severe neurologic conditions. An NGO-based children’s hospice with eight beds and a home visiting service is located in Almaty. A recently opened NGO-based Center for respiratory assistance and palliative care in Almaty provides high-quality services to children with neuromuscular diseases. In the absence of specialized services, children with terminal illnesses often occupy PICU beds, although the daily cost of an ICU bed is three times higher than that of a general ward bed [17]. Acute-care hospital beds are the most expensive choice for palliative care services, and thus, they should only be utilized for people with medical conditions that necessitate that degree of care [18]. Home-based services for terminally ill children still do not exist, except for the abovementioned Almaty children’s hospice. The need for establishing infrastructure for pediatric palliative care is of utmost importance. In 2021, 25,159 patients received palliative care in Kazakhstan, totaling 431,833 bed-days, or 17 bed-days per patient. There were 81 pediatric palliative care beds serving to 365 children in 2021. Table 1 summarizes the number of people with nine most common chronic diseases, their mortality, and the number of patients needing palliative care in Kazakhstan in 2021.

**TABLE 1:** Number of prevalent cases and deaths from diseases that are eligible for palliative care as well as number of patients which received palliative care in 2021 in Kazakhstan (National Research Center for Health Development) (Kazakhstan, 2020–2023).

Disease	Prevalent cases (total)	Number of deaths (adults)	Number of patients who received palliative care (adults) (n/%^a^)		Number of deaths (children) (n/%)	Number of patients who received palliative care (children) (n/%^a^)	
Cancer	190,159	14,049	3,180	23%	327	35	11%
Tuberculosis	10,186	296	157	53%	0	N/A	N/A
HIV	29,331	198	271	137%	5	N/A	N/A
Central nervous system	37,196	29,246	2,310	8%	610	267	44%
Cardiovascular	177,182	42,768	1,271	3%	337	14	4%
Liver	79,114	9,218	264	3%	37	1	3%
Alzheimer	494	298	2	1%	0	0	N/A
Respiratory	87,006	20,242	870	4%	457	3	1%
Diabetes	417,328	6,522	555	9%	16	1	6%
Total	1,027,670	122,837	8,880	7%	1,789	321	18%

^a^ Percentage of patients who received palliative care from the total number of deaths from the corresponding disease.

### Workforce

Currently there are 101 nurses and 45 physicians providing in-patient palliative care in hospices across Kazakhstan. The ratio of palliative physicians to palliative patients is 1:2,000, while the WHO recommends a ratio of 1 physician per 1,000 people in the general population [19]. In total, there are approximately 1,925 palliative beds in Kazakhstan, corresponding to national and international standards of 10 palliative beds per 100,000 people. However, 60% of all palliative beds are scattered throughout the vast country as single beds in regional hospitals, while, according to suggested standards, 80% of palliative beds should be in hospices and 20% in hospitals [20]. Correcting the balance of hospital versus hospice beds is critical for meeting the requirements of patients who cannot be cared for or who do not want to die at home [21]. To meet international standards, Kazakhstan should reorganize the wide distribution of single palliative beds in favor of new local hospices and palliative departments, which will contribute to the homogeneity of the quality of care and a smoother implementation of reforms in palliative care. A full list of bed distribution across regions of Kazakhstan is provided in Supplementary Appendix S1. It is worth mentioning that family caregivers represent a very important workforce in palliative care in Kazakhstan. The care for terminally ill patients in Kazakhstan lies entirely on the shoulders of families. Family caregivers’ active participation in providing care, even in inpatient settings, helps support patients’ quality-of-life and reduces the overall burden on medical staff and limited resources.

### Funding

Healthcare in Kazakhstan is funded by the Mandatory Social Health Insurance and by the government guaranteed Statutory Free Medical Assistance. The total palliative care budget amounted to approximately $6 million in 2020, $10 million in 2021, $15 million in 2022, and is projected to reach $22 million in 2023. Despite the substantial increase in the annual funding, it is important to ensure that the allocated budget is used efficiently and effectively to meet patients’ needs. Funding for palliative care is based on bed-day reimbursement, i.e., the length of stay of each patient, without considering actual costs of inpatient care. Table 2 shows costs of palliative care in different settings in Kazakhstan. Most consumables, such as diapers or oxygen concentrators, are mainly purchased out of pocket by families due to insufficient funding that does not match patients’ needs and lack of coordination with social security services.

**TABLE 2:** Nationally available palliative infrastructure and funding (National Research Center for Health Development) (Kazakhstan, 2020–2023).

	Settings	Cost per bed day ($)	Number of facilities	Total number of beds
1	Hospices	21	12	596
2	Palliative units	21	22	182
3	General hospitals^a^	Variable^b^	217	1,147
4	Mobile teams	14.5	209	

^a^ Has at least one palliative bed per hospital.

^b^ Depends on the budget and location of each hospital.

### Essential Palliative Care Medicines

**FIGURE 2:**
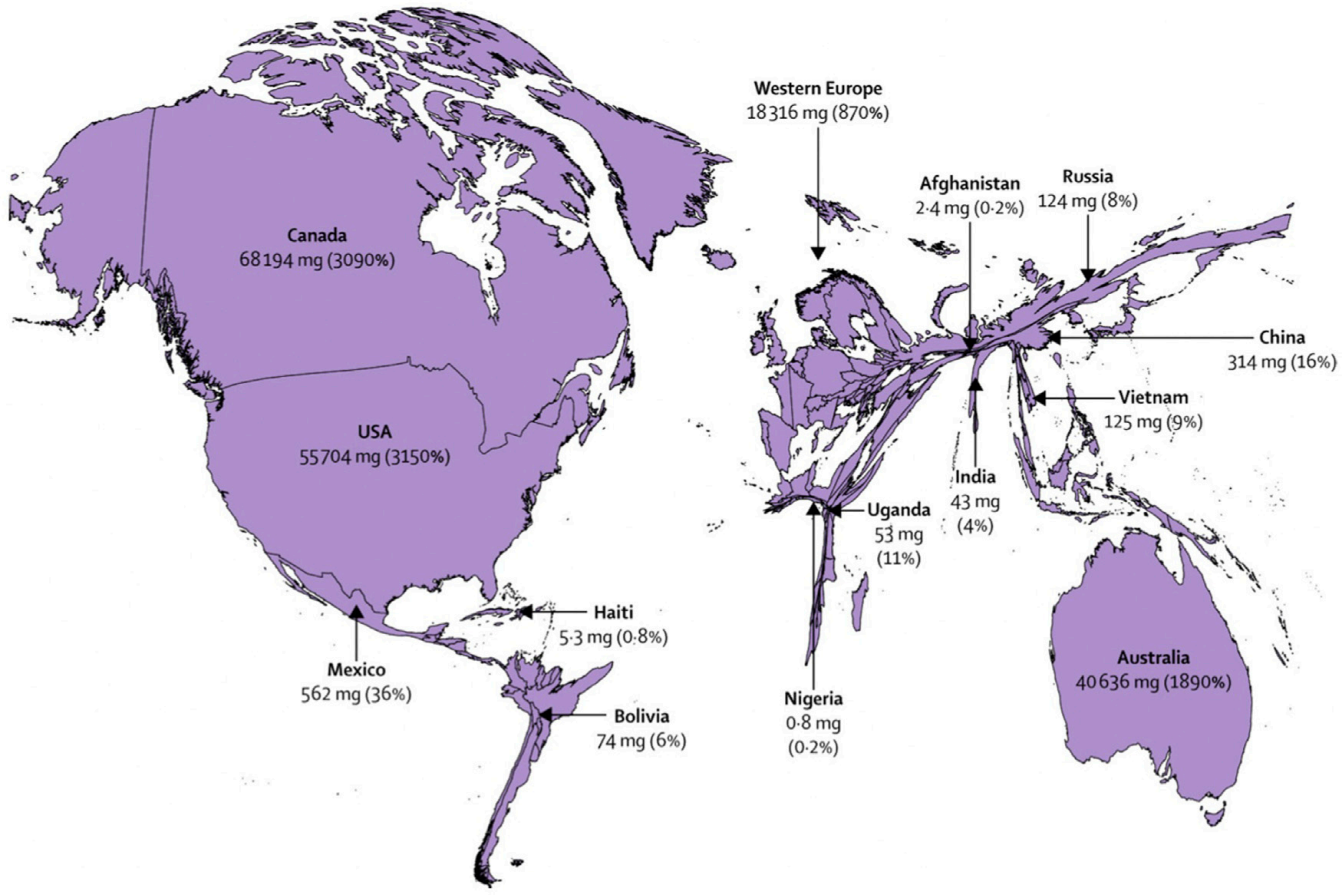
World distribution of morphine-equivalent opioids and the percentage of covered needs for pain relief [9]. License number: 5320150276782. Provided by the Elsevier and Copyright Clearance Center (United States, 2018).

The need and access to opioids is a tracer of overall availability of palliative care and pain relief [9]. Pain relief, a pillar of palliative care, was found to be the most lacking and inequitably distributed health intervention in the world, as 90% of the world population consumes only 11% of available opioids [9, 22]. Only 1% of 380 metric tons of morphine-equivalent opioids is distributed to low-income countries [23]. Figure 2 represents the world distribution of morphine-equivalent opioids (mg/patient), and percentage of pain relief needs that were met [9]. In 2018, the Worldwide Hospice Palliative Care Alliance estimated that the deficit of morphine-equivalent opioids in Kazakhstan was approximately 178 kgs [3]. At the same time, the Lancet Commission ranked Kazakhstan assessed that Kazakhstan covers 10% of needs for morphine, which is consistent an analysis conducted by KAPC which showed that up to 95% of deceased cancer patients in Kazakhstan died without receiving adequate pain management [2]. Moreover, the use of opioids for children in Kazakhstan is avoided, while adequate pain relief for one child costs less than $1/day [9]. In 2021, the Lancet Commission and the International Narcotics Control Board, ranked Kazakhstan 95th and 132nd country in opioid usage, respectively, with an average consumption of 1.31 mg/person, compared to 480.28 mg/person in Germany and 201.85 mg in Switzerland [22, 23]. By comparison, Kazakhstan consumes 15,000 and 68,000 times less morphine than in Belarus and in Canada, respectively [1, 9]. The state covers only 17 medications related to palliative care, while the list of necessary medicines of the International Association for Hospice and Palliative Care includes 33 medicines to control various symptoms such as depression, insomnia, pain, vomiting, etc. [13] However, between January and June of 2022, out of the 14,000 patients experiencing severe pain, only 7% were prescribed fentanyl patches, 10% morphine injections, and 83% tramadol. In Kazakhstan, like in many LMICs, patients who need morphine often have to seek admission to an in-patient facility in order to receive opioid pain relief [9].

### Education

Currently, there is no recognized specialty in “palliative care” for physicians and nurses in Kazakhstan. The only existing advanced training course (specialization), termed “hospice and palliative care,” is available solely for physicians, although nurses represent the main workforce in palliative care. In 2020, elective palliative care courses were introduced into educational programs for physicians, nurses, and paramedics for both undergraduate and postgraduate studies. However, the content, scope, and structure of such courses are non-standardized and often they are inconsistent with international requirements. While high-quality education requires trained faculty, there are no palliative care specialists who can provide such training.

## Policy Options and Recommendations

Recent developments have undoubtedly improved the image of Kazakhstan’s healthcare, however, to join the group of countries where palliative care is available, accessible, and affordable, Kazakhstan must integrate palliative services into the mainstream healthcare system at all levels throughout the country, from primary healthcare to hospices, and from major cities to remote villages [3]. Based on the existing evidence authors suggest that the palliative policy in Kazakhstan as well as other LMICs in similar settings should prioritize and focus on the following affordable steps:

• Establish continuous and transparent mechanisms of quantitative monitoring of quality of palliative care services and its outcomes.
• Establish registry of patients in need of palliative care, which is necessary for effective healthcare systems [1].
• Reorganize the distribution of palliative beds across regions in favor of established hospices and palliative care units.
• Remove system barriers to availability of opioids and close the gap between needs and actual consumption of opioids by expanding the list of available pain medication beyond injectable morphine and fentanyl patches.
• Establish regular courses for physicians on pain and symptom management and safe use of opioids.
• Provide educational opportunities for all healthcare providers and establishing palliative care specialization for nurses. The training required for healthcare providers to implement palliative care at each level of healthcare has been recommended by WHO and described in the literature [24].
• Establish mobile palliative care teams to improve access to palliative care in remote areas, where half of the population lives without access to palliative care [6].
• Examine perspectives and challenges of key stakeholders in palliative care (patients, families, healthcare professionals, providers, and policymakers) to identify and address gaps.
• Encourage awareness-building campaigns and public education regarding palliative care and pain relief, which is key to expanding access [9].
• Establish mobile teams for home-based care for patients whose palliative care needs could be met in their place of residence even after discharge. Should these patients require specialized inpatient care, they can be referred to a local hospice or palliative care unit.
• Prioritize oral forms of morphine over injections. Immediate-release oral morphine represents an effective, essential, and inexpensive intervention which is unjustifiably denied to most patients in need in LMICs.
• Integrate family caregivers into palliative care as they represent a major workforce and are crucially needed in LMICs settings. Empowering and utilizing them could improve the quality of care and reduce the burden on the resource-limited system.
• Participate in international agenda and collaborate with international stakeholders, such as the World Hospice Palliative Care Alliance and the International Palliative Care Association, which frequently offer programs and grants designed for LMICs facing similar challenges.
• Establish a cohort of palliative care specialists with advanced knowledge and skills to deliver high-quality palliative care services and provide training to others [1].
• Develop free online and onsite educational resources on palliative care for family caregivers can be an additional effective measure to improve the quality of life of patients and their families in resource-limited settings, whereas a wide range of medical staff should also have access to basic palliative training to make palliative literacy ubiquitous in all settings [25].

While goals of extending life are well-prioritized and well-funded, they need to be followed by goals to reduce suffering by offering adequate pain relief and symptom management at the end-of-life. Therefore, developing high-quality palliative care services must be prioritized by the Ministry of Health to ensure higher quality-of-life and end-of-life care among people with terminal diseases.

## Conclusion

In conclusion, the development of palliative care, in addition to the “ethical imperative” of eliminating the suffering of people with life-threatening illnesses, has several benefits for the state: it saves healthcare budgets by freeing up hospital beds, it reduces the number of hospitalizations and medical interventions, it reduces social tension and frees economically active family members, and improves society as a whole [26]. Being at the core of the universal health coverage, palliative care might be the least costly among all its components [1]. The COVID-19 pandemic set new obstacles for palliative care providers worldwide; however, patients cannot wait for proper changes to take place. Well-designed and appropriately financed palliative care relieves pressures on other parts of the health system and reduces overall costs [9].
