# Supplementary material for: Challenges for Developing Palliative Care Services in Resource- Limited Settings of Kazakhstan
Source: Public Health Rev. 2023 Aug 18;44:1605672. doi: 10.3389/phrs.2023.1605672 (PMC10476099; doi:10.3389/phrs.2023.1605672)
Supplement: Supplementary file 2 [file Table1.pdf]

Number of beds in the profile "Palliative care" in the context of the MOE and regions of the RK, according to the IS "RMS" as of December 31, 2021

№	Region	Medical organization	Bed Profile	Source of funding	Number of 24-hour inpatient beds
1	2	3	4	5	6
1	Akmola region	State communal enterprise on the right of economic management "Arshaly District Hospital" under the health department of Akmola region	Palliative care	Republican (BP 067, PP 100) "Transfers of the Federal Migration Service to pay for the state budget".	2
2	Akmola region	Akmola Regional Center of Phthisiopulmonology named after Konyratbek Kurmanbayev, a state municipal enterprise under the right of economic management under the Health Department of Akmola Region	Palliative care	Republican (BP 067, PP 100) Tuberculosis	2
3	Akmola region	State communal enterprise on the right of economic management "Zerenda district hospital" under the health department of Akmola region	Palliative care	Republican (BP 067, PP 100) "Transfers of the Federal Migration Service to pay for the state budget".	35
4	Akmola region	State municipal enterprise on the right of economic management "Kokshetau city multidisciplinary hospital" under the health department of Akmola region	Palliative care	Republican (BP 067, PP 100) "Transfers of the Federal Migration Service to pay for the state budget".	30
5	Akmola region	State communal enterprise on the right of economic management "Stepnogorsk multi-profile city hospital" under the health department of Akmola region	Palliative care	Republican (BP 067, PP 100) "Transfers of the Federal Migration Service to pay for the state budget".	18
6	Akmola region	State communal enterprise on the right of economic management "Akkol district hospital" under the health department of Akmola region	Palliative care	Republican (BP 067, PP 100) village	2
7	Akmola region	State communal enterprise on the right of economic management "Multidisciplinary regional hospital" under the health department of Akmola region	Palliative care	Republican (BP 067, PP 100) Oncology	19
8	Akmola region	State communal enterprise on the right of economic management "Multidisciplinary regional hospital" under the health department of Akmola region	Palliative care	Republican (BP 067, PP 100) "Transfers of the Federal Migration Service to pay for the state budget".	1
9	Aktobe region	Marat Ospanov West Kazakhstan Medical University non-profit joint-stock company	Palliative care	Republican (BP 067, PP 100) Oncology	20
10	Aktobe region	State communal enterprise "Alginskiy District Hospital" on the right of economic management of the state institution "Department of Health of Aktobe region".	Palliative care	Republican (BP 067, PP 100) village	1
11	Aktobe region	State communal enterprise "Aitekebi district hospital" on the right of economic management of the State Institution of the Department of Health of Aktobe region	Palliative care	Republican (BP 067, PP 100) village	1
12	Aktobe region	State communal enterprise Baiganinsky district hospital on the right of economic management of the State Institution of the Department of Health of Aktobe region	Palliative care	Republican (BP 067, PP 100) village	1
13	Aktobe region	State communal enterprise Irgiz District Hospital on the right of economic management State Institution Department of Health of Aktobe region	Palliative care	Republican (BP 067, PP 100) village	1
14	Aktobe region	State communal enterprise "Kargala District Hospital" on the right of economic management of the State Institution Department of Health of Aktobe region	Palliative care	Republican (BP 067, PP 100) village	1
15	Aktobe region	State communal enterprise "Martuk district hospital" on the right of economic management State Institution of the Department of Health of Aktobe region	Palliative care	Republican (BP 067, PP 100) village	1
16	Aktobe region	State communal enterprise "Mugalzhar district hospital" on the right of economic management of the State Institution Department of Health of Aktobe region	Palliative care	Republican (BP 067, PP 100) village	1
17	Aktobe region	State communal enterprise "Temir District Hospital" on the right of economic management of the State Institution of the Department of Health of Aktobe region	Palliative care	Republican (BP 067, PP 100) village	1
18	Aktobe region	State communal enterprise "Kobda district hospital" on the right of economic management State Institution Department of Health of Aktobe region	Palliative care	Republican (BP 067, PP 100) "Transfers of the Federal Migration Service to pay for the state budget".	1
19	Aktobe region	State communal enterprise "Khromtau district hospital" on the right of economic management of the State Institution of the Department of Health of Aktobe region	Palliative care	Republican (BP 067, PP 100) village	1
20	Aktobe region	State communal enterprise "Shalkar district hospital" on the right of economic management of the State Institution of the Department of Health of Aktobe region	Palliative care	Republican (BP 067, PP 100) village	1
21	Aktobe region	Limited Liability Partnership "Aktobe Railway Hospital	Palliative care	Republican (BP 067, PP 100) "Transfers of the Federal Migration Service to pay for the state budget".	1
22	Aktobe region	State communal enterprise "Emergency Medical Care Hospital" on the right of economic management of the State Institution Health Department of Aktobe region	Palliative care	Republican (BP 067, PP 100) "Transfers of the Federal Migration Service to pay for the state budget".	20

D
OC
24
ID
KZ
SI
U9
42
02
21
00
09
61
87
CD
E9
EF

23	Aktobe region	State communal enterprise "Center for Maternal and Child Health Protection" on the right of economic management of the state institution "Department of Health of Aktobe region".	Palliative care	Republican (BP 067, PP 100) "Transfers of the Federal Migration Service to pay for the state budget".	2
24	Almaty region	State communal enterprise on the right of economic management "Multidisciplinary regional children's hospital" state institution "Department of Health Almaty region"	Palliative care	Republican (BP 067, PP 100) "Transfers of the Federal Migration Service to pay for the state budget".	2
25	Almaty region	State communal enterprise on the right of economic management Aksu Central District Hospital of the State Institution Department of Health of the Almaty Region Akimat of the Almaty Region	Palliative care	Republican (BP 067, PP 100) village	1
26	Almaty region	State communal enterprise on the right of economic management Aksu Central District Hospital of the State Institution Department of Health of the Almaty Region Akimat of the Almaty Region	Palliative care	Republican (BP 067, PP 100) village	1
27	Almaty region	Communal state enterprise on the right of economic management "Enbekshikazakh Multi-district Interdistrict Hospital" state institution "Department of Health of the Almaty region	Palliative care	Republican (BP 067, PP 100) village	5
28	Almaty region	State communal enterprise on the right of economic management "Sarkand Central District Hospital" state institution "Department of Health of the Almaty Region" Akimat of the Almaty region	Palliative care	Republican (BP 067, PP 100) village	1
29	Almaty region	State municipal enterprise on the right of economic management "Alakol Central District Hospital" State Institution "Department of Health of Almaty Region" Akimat of Almaty region	Palliative care	Republican (BP 067, PP 100) village	1
30	Almaty region	State municipal enterprise on the right of economic management "Regional Multidisciplinary Clinic" of the state institution "Department of Health of the Almaty region" Akimat of the Almaty region	Palliative care	Republican (BP 067, PP 100) "Transfers of the Federal Migration Service to pay for the state budget".	20
31	Almaty region	Public utility enterprise on the right of economic management "Karasai multi-district interdistrict hospital" state institution "Department of Health of the Almaty region"	Palliative care	Republican (BP 067, PP 100) village	2
32	Almaty region	Panfilov Multidisciplinary Interdistrict Hospital Communal State Enterprise on the right of economic management of the State Institution "Health Care Administration of Almaty Region	Palliative care	Republican (BP 067, PP 100) village	1
33	Almaty region	State communal enterprise on the right of economic management "Talgar central district hospital" state institution "Department of Health of Almaty region"	Palliative care	Republican (BP 067, PP 100) village	1
34	Almaty region	Communal State Enterprise on the right of economic management "Almaty Regional Multidisciplinary Clinic" State Institution "Department of Health of the Almaty region Akimat of Almaty region	Palliative care	Republican (BP 067, PP 100) "Transfers of the Federal Migration Service to pay for the state budget".	25
35	Almaty region	State communal enterprise on the right of economic management "District Hospital" of Boraldai village of Iliysk district of the state institution "Department of Health of the Almaty region".	Palliative care	Republican (BP 067, PP 100) village	2
36	Almaty region	Communal State Enterprise "Palliative Care Hospital of Almaty Oblast" State Institution "Health Care Administration of Almaty Oblast"	Palliative care	Republican (BP 067, PP 100) "Transfers of the Federal Migration Service to pay for the state budget".	35
37	Almaty region	State communal enterprise on the right of economic management "Almaty Regional Children's Clinical Hospital" state institution "Department of Health of Almaty Region"	Palliative care	Republican (BP 067, PP 100) "Transfers of the Federal Migration Service to pay for the state budget".	1
38	Almaty region	State communal enterprise on the right of economic management "Almaty Regional Children's Clinical Hospital" State Institution "Department of Health of Almaty Region"	Palliative care	Republican (BP 067, PP 100) "Transfers of the Federal Migration Service to pay for the state budget".	1
39	Almaty region	State communal enterprise on the right of economic management "Almaty Regional Children's Clinical Hospital" State Institution "Department of Health of Almaty Region"	Palliative care	Republican (BP 067, PP 100) "Transfers of the Federal Migration Service to pay for the state budget".	1
40	Atyrau region	Communal State Enterprise on the right of economic management "Atyrau Regional Hospital" Department of Health of Atyrau region	Palliative care	Republican (BP 067, PP 100) "Transfers of the Federal Migration Service to pay for the state budget".	10
41	Atyrau region	Communal State Enterprise on the right of economic management "Atyrau Regional Children's Hospital" Health Department of Atyrau region".	Palliative care	Republican (BP 067, PP 100) "Transfers of the Federal Migration Service to pay for the state budget".	2
42	Atyrau region	Communal state enterprise on the right of economic management "Atyrau Regional Oncological Dispensary Management of Health of Atyrau region".	Palliative care	Republican (BP 067, PP 100) "Transfers of the Federal Migration Service to pay for the state budget".	10
43	Atyrau region	Communal State Enterprise on the right of economic management "Inder district hospital" Health Department of Atyrau region	Palliative care	Republican (BP 067, PP 100) "Transfers of the Federal Migration Service to pay for the state budget".	1
44	Atyrau region	The Makat District Hospital Communal State Enterprise on the right of economic management of the Department of Health of Atyrau region	Palliative care	Republican (BP 067, PP 100) "Transfers of the Federal Migration Service to pay for the state budget".	10
45	Atyrau region	Communal state enterprise on the right of economic management "Atyrau regional hospital № 2" Department of Health of Atyrau region	Palliative care	Assets of the Fund on OSMS	2
46	Atyrau region	Communal state enterprise on the right of economic management "Atyrau regional hospital № 2" Department of Health of Atyrau region	Palliative care	Republican (BP 067, PP 100) "Transfers of the Federal Migration Service to pay for the state budget".	1
47	West Kazakhstan region	State communal enterprise on the right of economic management "Regional Oncological Dispensary" of the Department of Health Akimat of West Kazakhstan region	Palliative care	Republican (BP 067, PP 100) "Transfers of the Federal Migration Service to pay for the state budget".	25
48	West Kazakhstan region	State communal enterprise on the right of economic management "Akzhaik district hospital" Health Department of the Akimat of West Kazakhstan region	Palliative care	Republican (BP 067, PP 100) "Transfers of the Federal Migration Service to pay for the state budget".	1
49	West Kazakhstan region	State communal enterprise on the right of economic management "Akzhaik district hospital" Health Department of the Akimat of West Kazakhstan region	Palliative care	Republican (BP 067, PP 100) "Transfers of the Federal Migration Service to pay for the state budget".	1

50	West Kazakhstan region	State communal enterprise on the right of economic management "Akzhaik district hospital" Health Department of the Akimat of West Kazakhstan region	Palliative care	Republican (BP 067, PP 100) "Transfers of the Federal Migration Service to pay for the state budget".	1
51	West Kazakhstan region	State communal enterprise on the right of economic management "Bokeiorda district hospital" Health Department of the Akimat of West Kazakhstan region	Palliative care	Republican (BP 067, PP 100) "Transfers of the Federal Migration Service to pay for the state budget".	2
52	West Kazakhstan region	State communal enterprise on the right of economic management "Burlinsk District Hospital" Health Department of the Akimat of West Kazakhstan region	Palliative care	Republican (BP 067, PP 100) "Transfers of the Federal Migration Service to pay for the state budget".	2
53	West Kazakhstan region	State communal enterprise on the right of economic management "Zhangala District Hospital" Health Department of the Akimat of West Kazakhstan region	Palliative care	Republican (BP 067, PP 100) "Transfers of the Federal Migration Service to pay for the state budget".	1
54	West Kazakhstan region	State communal enterprise on the right of economic management "Zhanibek district hospital" Health Department of the Akimat of West Kazakhstan region	Palliative care	Republican (BP 067, PP 100) "Transfers of the Federal Migration Service to pay for the state budget".	1
55	West Kazakhstan region	State communal enterprise on the right of economic management "Hospital of Baiterek district" health department of the akimat of the West Kazakhstan region	Palliative care	Republican (BP 067, PP 100) "Transfers of the Federal Migration Service to pay for the state budget".	18
56	West Kazakhstan region	State communal enterprise on the right of economic management "2 Hospital of Baiterek district" Health Department of the Akimat of West Kazakhstan region	Palliative care	Republican (BP 067, PP 100) "Transfers of the Federal Migration Service to pay for the state budget".	5
57	West Kazakhstan region	State communal enterprise on the right of economic management "Karatobe district hospital" Health Department of the Akimat of West Kazakhstan region	Palliative care	Republican (BP 067, PP 100) "Transfers of the Federal Migration Service to pay for the state budget".	1
58	West Kazakhstan region	State communal enterprise on the right of economic management "Kaztalovsky district hospital" of the health department of the akimat of the West Kazakhstan region	Palliative care	Republican (BP 067, PP 100) "Transfers of the Federal Migration Service to pay for the state budget".	1
59	West Kazakhstan region	State communal enterprise on the right of economic management "2 Hospital of Kaztalovsky district" Health Department of the Akimat of West Kazakhstan region	Palliative care	Republican (BP 067, PP 100) "Transfers of the Federal Migration Service to pay for the state budget".	1
60	West Kazakhstan region	State communal enterprise on the right of economic management "Syrym District Hospital" Health Department of the Akimat of West Kazakhstan region	Palliative care	Republican (BP 067, PP 100) "Transfers of the Federal Migration Service to pay for the state budget".	19
61	West Kazakhstan region	State communal enterprise on the right of economic management "Taskala District Hospital" Health Department of the Akimat of West Kazakhstan region	Palliative care	Republican (BP 067, PP 100) "Transfers of the Federal Migration Service to pay for the state budget".	15
62	West Kazakhstan region	State communal enterprise on the right of economic management "Taskala District Hospital" Health Department of the Akimat of West Kazakhstan region	Palliative care	Republican (BP 067, PP 100) "Transfers of the Federal Migration Service to pay for the state budget".	1
63	West Kazakhstan region	State communal enterprise on the right of economic management "2-hospital of Terektinsky district" of the health department of the akimat of the West Kazakhstan region	Palliative care	Republican (BP 067, PP 100) "Transfers of the Federal Migration Service to pay for the state budget".	21
64	West Kazakhstan region	State communal enterprise on the right of economic management "Terektinsky district hospital" Health Department of the Akimat of West Kazakhstan region	Palliative care	Republican (BP 067, PP 100) "Transfers of the Federal Migration Service to pay for the state budget".	1
65	West Kazakhstan region	State communal enterprise on the right of economic management "Chingirlau district hospital" of the health department of the akimat of the West Kazakhstan region	Palliative care	Republican (BP 067, PP 100) "Transfers of the Federal Migration Service to pay for the state budget".	1
66	West Kazakhstan region	State communal enterprise on the right of economic management "Regional children's multidisciplinary hospital" Health Department of the Akimat of West Kazakhstan region	Palliative care	Republican (BP 067, PP 100) "Transfers of the Federal Migration Service to pay for the state budget".	1
67	West Kazakhstan region	State communal enterprise on the right of economic management "Regional children's multidisciplinary hospital" Health Department of the Akimat of West Kazakhstan region	Palliative care	Republican (BP 067, PP 100) "Transfers of the Federal Migration Service to pay for the state budget".	1
68	West Kazakhstan region	State communal enterprise on the right of economic management "Regional children's multidisciplinary hospital" Health Department of the Akimat of West Kazakhstan region	Palliative care	Republican (BP 067, PP 100) "Transfers of the Federal Migration Service to pay for the state budget".	2
69	West Kazakhstan region	State communal enterprise on the right of economic management "Regional children's multidisciplinary hospital" Health Department of the Akimat of West Kazakhstan region	Palliative care	Republican (BP 067, PP 100) "Transfers of the Federal Migration Service to pay for the state budget".	1
70	West Kazakhstan region	State communal enterprise on the right of economic management "Regional Center of Phthisiopulmonology" of the Department of Health of the Akimat of West Kazakhstan region	Palliative care	Republican (BP 067, PP 100) Tuberculosis	25
71	West Kazakhstan region	State communal enterprise on the right of economic management "Regional multidisciplinary hospital" of the health department of the akimat of the West Kazakhstan region	Palliative care	Republican (BP 067, PP 100) "Transfers of the Federal Migration Service to pay for the state budget".	2
72	West Kazakhstan region	State communal enterprise on the right of economic management "Regional multidisciplinary hospital" of the health department of the akimat of the West Kazakhstan region	Palliative care	Republican (BP 067, PP 100) "Transfers of the Federal Migration Service to pay for the state budget".	1
73	West Kazakhstan region	State communal enterprise on the right of economic management "Regional multidisciplinary hospital" of the health department of the akimat of the West Kazakhstan region	Palliative care	Republican (BP 067, PP 100) "Transfers of the Federal Migration Service to pay for the state budget".	2

D
OC
24
ID
KZ
SI
U9
42
02
21
00
09
61
87
CD
E9
EF

74	West Kazakhstan region	State communal enterprise on the right of economic management "Regional multidisciplinary hospital" of the health department of the akimat of the West Kazakhstan region	Palliative care	Republican (BP 067, PP 100) "Transfers of the Federal Migration Service to pay for the state budget".	2
75	West Kazakhstan region	State communal enterprise on the right of economic management "Regional multidisciplinary hospital" of the health department of the akimat of the West Kazakhstan region	Palliative care	Republican (BP 067, PP 100) "Transfers of the Federal Migration Service to pay for the state budget".	7
76	West Kazakhstan region	State communal enterprise on the right of economic management "Regional multidisciplinary hospital" of the health department of the akimat of the West Kazakhstan region	Palliative care	Republican (BP 067, PP 100) "Transfers of the Federal Migration Service to pay for the state budget".	2
77	West Kazakhstan region	Uniserv Medical Center Limited Liability Partnership	Palliative care	Self-accounting	2
78	Zhambyl region	Communal State Enterprise on the right of economic management "Central District Hospital of Bayzak District" Health Department of Zhambyl Oblast Akimat	Palliative care	Republican (BP 067, PP 100) "Transfers of the Federal Migration Service to pay for the state budget".	2
79	Zhambyl region	Communal State Enterprise on the right of economic management "Central District Hospital of Zhambyl district" Health Department of Zhambyl Region Akimat	Palliative care	Republican (BP 067, PP 100) "Transfers of the Federal Migration Service to pay for the state budget".	10
80	Zhambyl region	State communal enterprise on the right of economic management "Central district hospital of Zhualy district" Health Department of Zhambyl Oblast Akimat	Palliative care	Republican (BP 067, PP 100) "Transfers of the Federal Migration Service to pay for the state budget".	3
81	Zhambyl region	State communal enterprise on the right of economic management "Korday Central District Hospital" Health Department of the Akimat of Zhambyl Region	Palliative care	Republican (BP 067, PP 100) "Transfers of the Federal Migration Service to pay for the state budget".	3
82	Zhambyl region	State communal enterprise on the right of economic management "Central district hospital of Merken district" Department of Health of the Akimat of Zhambyl region	Palliative care	Republican (BP 067, PP 100) "Transfers of the Federal Migration Service to pay for the state budget".	2
83	Zhambyl region	State communal enterprise on the right of economic management "Central district hospital of Merken district" Department of Health of the Akimat of Zhambyl region	Palliative care	Republican (BP 067, PP 100) "Transfers of the Federal Migration Service to pay for the state budget".	2
84	Zhambyl region	Communal State Enterprise on the right of economic management "Central District Hospital of Moyinkum district of the Department of Health Akimat of Zhambyl region".	Palliative care	Republican (BP 067, PP 100) village	2
85	Zhambyl region	Communal State Enterprise on the right of economic management "Central District Hospital of the district named after T. Ryskulov" Department of Health Akimat of Zhambyl region	Palliative care	Republican (BP 067, PP 100) "Transfers of the Federal Migration Service to pay for the state budget".	4
86	Zhambyl region	Communal State Enterprise on the right of economic management "Central District Hospital of Sarysu district" Health Department of Zhambyl Oblast Akimat	Palliative care	Republican (BP 067, PP 100) "Transfers of the Federal Migration Service to pay for the state budget".	3
87	Zhambyl region	Communal State Enterprise on the right of economic management "Central District Hospital of Sarysu district" Health Department of Zhambyl Oblast Akimat	Palliative care	Republican (BP 067, PP 100) "Transfers of the Federal Migration Service to pay for the state budget".	1
88	Zhambyl region	Communal State Enterprise on the right of economic management "Central District Hospital of Sarysu district" Health Department of Zhambyl Oblast Akimat	Palliative care	Republican (BP 067, PP 100) "Transfers of the Federal Migration Service to pay for the state budget".	3
89	Zhambyl region	Communal State Enterprise on the right of economic management "Central District Hospital of Sarysu district" Health Department of Zhambyl Oblast Akimat	Palliative care	Republican (BP 067, PP 100) "Transfers of the Federal Migration Service to pay for the state budget".	2
90	Zhambyl region	Communal State Enterprise on the right of economic management "Central District Hospital of Talas District Department of Health Akimat of Zhambyl Region".	Palliative care	Republican (BP 067, PP 100) "Transfers of the Federal Migration Service to pay for the state budget".	3
91	Zhambyl region	State communal enterprise on the right of economic management "Shuskaya Central District Hospital" Health Department of the Akimat of Zhambyl Region	Palliative care	Republican (BP 067, PP 100) "Transfers of the Federal Migration Service to pay for the state budget".	2
92	Zhambyl region	Farm-Ay Limited Liability Partnership	Palliative care	Republican (BP 067, PP 100) "Transfers of the Federal Migration Service to pay for the state budget".	40
93	Zhambyl region	Communal State Enterprise on the right of economic ownership "Zhambyl Regional Center of Phthisiopulmonology Department of Health Akimat of Zhambyl region".	Palliative care	Republican (BP 067, PP 100) Tuberculosis	3
94	Zhambyl region	State communal enterprise on the right of economic management "Municipal multi-profile hospital of the Department of Health Akimat of Zhambyl region".	Palliative care	Republican (BP 067, PP 100) "Transfers of the Federal Migration Service to pay for the state budget".	36
95	Zhambyl region	State communal enterprise on the right of economic management "Zhambyl Regional Multidisciplinary Children's Hospital" Health Department of the Akimat of Zhambyl Region".	Palliative care	Republican (BP 067, PP 100) "Transfers of the Federal Migration Service to pay for the state budget".	10
96	Karaganda region	Municipal State Enterprise "Multiprofile Hospital №1 of Karaganda city" of the Health Department of Karaganda region	Palliative care	Republican (BP 067, PP 100) "Transfers of the Federal Migration Service to pay for the state budget".	8
97	Karaganda region	Communal State Enterprise "Children's Hospital of Karaganda city" Health Department of Karaganda region	Palliative care	Republican (BP 067, PP 100) "Transfers of the Federal Migration Service to pay for the state budget".	7
98	Karaganda region	Communal State Enterprise on the right of economic management "Multidisciplinary Hospital named after Professor H.Zh. Makazhanov" Health Department of Karaganda region	Palliative care	Republican (BP 067, PP 100) "Transfers of the Federal Migration Service to pay for the state budget".	5

D
OC
24
ID
KZ
SI
U9
42
02
21
00
09
61
87
CD
E9
EF

99	Karaganda region	Communal State Enterprise "Central Hospital №1 of Satpayev" Health Department of Karaganda region	Palliative care	Republican (BP 067, PP 100) "Transfers of the Federal Migration Service to pay for the state budget".	15
100	Karaganda region	Municipal State Enterprise "Multidisciplinary Center for Mothers and Children of Temirtau" Health Department of Karaganda region	Palliative care	Republican (BP 067, PP 100) "Transfers of the Federal Migration Service to pay for the state budget".	5
101	Karaganda region	Communal State Enterprise "Central Hospital of Shakhtinsk" Akimat of Karaganda Region Health Department of Karaganda Region	Palliative care	Republican (BP 067, PP 100) "Transfers of the Federal Migration Service to pay for the state budget".	20
102	Karaganda region	Communal State Enterprise "Central Hospital of Shakhtinsk" Akimat of Karaganda Region Health Department of Karaganda Region	Palliative care	Republican (BP 067, PP 100) "Transfers of the Federal Migration Service to pay for the state budget".	2
103	Karaganda region	Communal State Enterprise "Central Hospital of Saran" Health Department of Karaganda region	Palliative care	Republican (BP 067, PP 100) "Transfers of the Federal Migration Service to pay for the state budget".	7
104	Karaganda region	Limited Liability Partnership Hippocrates Medical Firm	Palliative care	Republican (BP 067, PP 100) "Transfers of the Federal Migration Service to pay for the state budget".	35
105	Karaganda region	Limited Liability Partnership "Clinic "Zhansaya"	Palliative care	Republican (BP 067, PP 100) "Transfers of the Federal Migration Service to pay for the state budget".	50
106	Kostanay region	Communal State Enterprise "Altynsarin District Hospital" Health Department of the Kostanai Region Akimat	Palliative care	Republican (BP 067, PP 100) village	1
107	Kostanay region	Communal State Enterprise "Altynsarin District Hospital" Health Department of the Kostanai Region Akimat	Palliative care	Republican (BP 067, PP 100) "Transfers of the Federal Migration Service to pay for the state budget".	5
108	Kostanay region	Amangeldy District Hospital Communal State Enterprise of the Health Department of the Kostanai Region Akimat	Palliative care	Republican (BP 067, PP 100) "Transfers of the Federal Migration Service to pay for the state budget".	6
109	Kostanay region	Communal State Enterprise "Auliekol District Hospital" Health Department of the akimat of Kostanai region	Palliative care	Republican (BP 067, PP 100) "Transfers of the Federal Migration Service to pay for the state budget".	30
110	Kostanay region	Communal State Enterprise "Kushmurun rural hospital" Health Department of the akimat of Kostanai region	Palliative care	Republican (BP 067, PP 100) "Transfers of the Federal Migration Service to pay for the state budget".	5
111	Kostanay region	Communal State Enterprise "Denisovsky district hospital" Health Department of the akimat of the Kostanai region	Palliative care	Republican (BP 067, PP 100) "Transfers of the Federal Migration Service to pay for the state budget".	10
112	Kostanay region	Dzhangeldy District Hospital Communal State Enterprise of the Health Department of the Kostanai Region Akimat	Palliative care	Republican (BP 067, PP 100) "Transfers of the Federal Migration Service to pay for the state budget".	6
113	Kostanay region	Communal State Enterprise "Zhitikarinsky district hospital" Health Department of the akimat of the Kostanai region	Palliative care	Republican (BP 067, PP 100) village	1
114	Kostanay region	Communal State Enterprise "Zhitikarinsky district hospital" Health Department of the akimat of the Kostanai region	Palliative care	Republican (BP 067, PP 100) "Transfers of the Federal Migration Service to pay for the state budget".	4
115	Kostanay region	Kamystinskaya District Hospital Communal State Enterprise of the Health Department of the Kostanai Region Akimat	Palliative care	Republican (BP 067, PP 100) "Transfers of the Federal Migration Service to pay for the state budget".	5
116	Kostanay region	Communal State Enterprise "Karabalyk District Hospital" Health Department of the akimat of the Kostanai region	Palliative care	Republican (BP 067, PP 100) "Transfers of the Federal Migration Service to pay for the state budget".	15
117	Kostanay region	State public enterprise "Karasu district hospital" Health Department of the akimat of the Kostanai region	Palliative care	Republican (BP 067, PP 100) "Transfers of the Federal Migration Service to pay for the state budget".	6
118	Kostanay region	Communal State Enterprise "Oktyabrskaya Rural Hospital" Health Department of the Kostanai Region Akimat	Palliative care	Republican (BP 067, PP 100) "Transfers of the Federal Migration Service to pay for the state budget".	4
119	Kostanay region	Kostanay Rayon Hospital Communal State Enterprise of the Health Department of the Kostanay Region Akimat	Palliative care	Republican (BP 067, PP 100) "Transfers of the Federal Migration Service to pay for the state budget".	3
120	Kostanay region	Mendykara District Hospital Communal State Enterprise of the Health Department of the Kostanai Region Akimat	Palliative care	Republican (BP 067, PP 100) "Transfers of the Federal Migration Service to pay for the state budget".	5
121	Kostanay region	Communal State Enterprise "Naurzum district hospital" Health Department of the akimat of Kostanai region	Palliative care	Republican (BP 067, PP 100) "Transfers of the Federal Migration Service to pay for the state budget".	15
122	Kostanay region	Sarykol District Hospital Communal State Enterprise of the Health Department of the Kostanai Region Akimat	Palliative care	Republican (BP 067, PP 100) "Transfers of the Federal Migration Service to pay for the state budget".	5
123	Kostanay region	Communal State Enterprise "District Hospital of Beimbet Mailin District" Health Department of the akimat of Kostanai region	Palliative care	Republican (BP 067, PP 100) "Transfers of the Federal Migration Service to pay for the state budget".	2
124	Kostanay region	Communal State Enterprise "Uzunkol district hospital" Health Department of the akimat of Kostanai region	Palliative care	Republican (BP 067, PP 100) "Transfers of the Federal Migration Service to pay for the state budget".	10
125	Kostanay region	Fyodorovsky District Hospital Communal State Enterprise of the Health Department of the Kostanai Region Akimat	Palliative care	Republican (BP 067, PP 100) "Transfers of the Federal Migration Service to pay for the state budget".	4
126	Kostanay region	Kostanay City Hospital Communal State Enterprise of the Health Department of the Kostanay Region Akimat	Palliative care	Republican (BP 067, PP 100) "Transfers of the Federal Migration Service to pay for the state budget".	10
127	Kostanay region	Municipal State Enterprise "Arkalyk Regional Hospital" Health Department of the Kostanai Region Akimat	Palliative care	Republican (BP 067, PP 100) "Transfers of the Federal Migration Service to pay for the state budget".	15
128	Kostanay region	Communal State Enterprise "Kachar City Hospital" Department of Health Akimat of Kostanay region	Palliative care	Republican (BP 067, PP 100) "Transfers of the Federal Migration Service to pay for the state budget".	10
129	Kostanay region	Communal State Enterprise "Lisakovsky City Hospital" Health Department of the akimat of the Kostanai region	Palliative care	Republican (BP 067, PP 100) "Transfers of the Federal Migration Service to pay for the state budget".	5
130	Kostanay region	Kostanay Regional Children's Hospital Communal State Enterprise of the Health Department of the Kostanay Region Akimat	Palliative care	Republican (BP 067, PP 100) "Transfers of the Federal Migration Service to pay for the state budget".	3
131	Kostanay region	Kostanay City Oncological Multiprofile Hospital, a municipal state enterprise of the Department of Health of the Kostanay Region Akimat	Palliative care	Republican (BP 067, PP 100) "Transfers of the Federal Migration Service to pay for the state budget".	20

D
OC
24
ID
KZ
SI
U9
42
02
21
00
09
61
87
CD
E9
EF

132	Kyzylorda region	Communal state enterprise on the right of economic management "Multidisciplinary Regional Hospital" Health Department of Kyzylorda Oblast	Palliative care	Republican (BP 067, PP 100) "Transfers of the Federal Migration Service to pay for the state budget".	20
133	Kyzylorda region	Communal State Enterprise on the right of economic management "Multidisciplinary Regional Children's Hospital" Health Department of Kyzylorda region	Palliative care	Republican (BP 067, PP 100) "Transfers of the Federal Migration Service to pay for the state budget".	5
134	Kyzylorda region	Communal state enterprise on the right of economic management "Kyzylorda Regional Cancer Center" Health Department of Kyzylorda Region	Palliative care	Republican (BP 067, PP 100) "Transfers of the Federal Migration Service to pay for the state budget".	30
135	Kyzylorda region	Kazalinsk Railway Hospital Limited Liability Partnership	Palliative care	Republican (BP 067, PP 100) "Transfers of the Federal Migration Service to pay for the state budget".	5
136	Kyzylorda region	Communal state enterprise on the right of economic management "Aralsk Interdistrict Hospital" of the Health Department of Kyzylorda Oblast	Palliative care	Republican (BP 067, PP 100) "Transfers of the Federal Migration Service to pay for the state budget".	5
137	Kyzylorda region	Communal state enterprise on the right of economic management "Kazaly Interdistrict Hospital" Health Department of Kyzylorda Oblast	Palliative care	Assets of the Fund on OSMS	1
138	Kyzylorda region	Communal state enterprise on the right of economic management "Kazaly Interdistrict Hospital" Health Department of Kyzylorda Oblast	Palliative care	Assets of the Fund on OSMS	1
139	Kyzylorda region	Communal state enterprise on the right of economic management "Kazaly Interdistrict Hospital" Health Department of Kyzylorda Oblast	Palliative care	Republican (BP 067, PP 100) "Transfers of the Federal Migration Service to pay for the state budget".	6
140	Kyzylorda region	Communal state enterprise on the right of economic management "Karmaksha district hospital" Health Department of Kyzylorda region	Palliative care	Republican (BP 067, PP 100) "Transfers of the Federal Migration Service to pay for the state budget".	5
141	Kyzylorda region	Communal state enterprise on the right of economic management "Zhalagash District Hospital" Health Department of Kyzylorda Region	Palliative care	Republican (BP 067, PP 100) "Transfers of the Federal Migration Service to pay for the state budget".	10
142	Kyzylorda region	Communal state enterprise on the right of economic management "Syrdarya District Hospital" Health Department of Kyzylorda Region	Palliative care	Republican (BP 067, PP 100) "Transfers of the Federal Migration Service to pay for the state budget".	10
143	Kyzylorda region	Communal state enterprise on the right of economic management "Shiyeli Interdistrict Hospital" Health Department of Kyzylorda Oblast	Palliative care	Republican (BP 067, PP 100) "Transfers of the Federal Migration Service to pay for the state budget".	5
144	Kyzylorda region	Communal state enterprise on the right of economic management "Zhanakorgan Interdistrict Hospital" Health Department of Kyzylorda Region	Palliative care	Republican (BP 067, PP 100) "Transfers of the Federal Migration Service to pay for the state budget".	10
145	Mangistau region	State communal enterprise on the right of economic management "Mangistau regional multidisciplinary hospital" Health Department of Mangistau region	Palliative care	Republican (BP 067, PP 100) "Transfers of the Federal Migration Service to pay for the state budget".	15
146	Mangistau region	State communal enterprise on the right of economic management "Regional Oncological Dispensary" of the Department of Health of Mangistau region	Palliative care	Republican (BP 067, PP 100) "Transfers of the Federal Migration Service to pay for the state budget".	25
147	Mangistau region	State communal enterprise on the right of economic management "Mangistau Central District Hospital" Health Department of Mangistau region	Palliative care	Republican (BP 067, PP 100) village	2
148	Mangistau region	State communal enterprise on the right of economic management "Mangistau Central District Hospital" Health Department of Mangistau region	Palliative care	Republican (BP 067, PP 100) village	1
149	Mangistau region	State communal enterprise on the right of economic management "Tupkaragan central district hospital" Health Department of Mangistau region akimat of Mangistau region	Palliative care	Republican (BP 067, PP 100) village	1
150	Mangistau region	Limited Liability Partnership "Medical Center of Cardiology and Internal Medicine "ARCHIMED	Palliative care	Republican (BP 067, PP 100) "Transfers of the Federal Migration Service to pay for the state budget".	20
151	Pavlodar region	Pavlodar Regional Children's Hospital, Pavlodar Oblast Health Department, Pavlodar Oblast Akimat, is a communal state enterprise under the right of economic management	Palliative care	Republican (BP 067, PP 100) "Transfers of the Federal Migration Service to pay for the state budget".	1
152	Pavlodar region	Pavlodar Regional Children's Hospital, Pavlodar Oblast Health Department, Pavlodar Oblast Akimat, is a communal state enterprise under the right of economic management	Palliative care	Republican (BP 067, PP 100) "Transfers of the Federal Migration Service to pay for the state budget".	1
153	Pavlodar region	Communal State Enterprise on the right of economic management "City Hospital of Aksu" Department of Health of Pavlodar region, akimat of Pavlodar region	Palliative care	Republican (BP 067, PP 100) "Transfers of the Federal Migration Service to pay for the state budget".	5
154	Pavlodar region	Communal State Enterprise on the right of economic management "Aktogai district hospital" Department of Health of the Pavlodar region, the akimat of the Pavlodar region	Palliative care	Republican (BP 067, PP 100) "Transfers of the Federal Migration Service to pay for the state budget".	2
155	Pavlodar region	Communal State Enterprise on the right of economic management "Bayanaul district hospital" Health Department of Pavlodar region, the akimat of the Pavlodar region	Palliative care	Republican (BP 067, PP 100) village	1
156	Pavlodar region	Communal State Enterprise on the right of economic management "Zhelezinskaya district hospital" Health Department of Pavlodar region, akimat of Pavlodar region	Palliative care	Republican (BP 067, PP 100) "Transfers of the Federal Migration Service to pay for the state budget".	1

D
OC
24
ID
KZ
SI
U9
42
02
21
00
09
61
87
CD
E9
EF

157	Pavlodar region	Communal State Enterprise on the right of economic management "Terekol District Hospital" Health Department of Pavlodar region, Pavlodar region akimat	Palliative care	Republican (BP 067, PP 100) village	1
158	Pavlodar region	Communal State Enterprise on the right of economic management "May district hospital" Health Department of Pavlodar region, the akimat of the Pavlodar region	Palliative care	Republican (BP 067, PP 100) village	2
159	Pavlodar region	Utility state enterprise on the right of economic management "Assumption district hospital" Department of Health of the Pavlodar region, the akimat of the Pavlodar region	Palliative care	Republican (BP 067, PP 100) "Transfers of the Federal Migration Service to pay for the state budget".	1
160	Pavlodar region	Communal State Enterprise on the right of economic management "Shcherbakta District Hospital" Health Department of Pavlodar Region, Akimat of Pavlodar region	Palliative care	Republican (BP 067, PP 100) "Transfers of the Federal Migration Service to pay for the state budget".	1
161	Pavlodar region	Pavlodar Regional Phthisiopulmonology Center, Pavlodar Oblast Health Department, Pavlodar Oblast Akimat State Municipal Enterprise	Palliative care	Republican (BP 067, PP 100) Tuberculosis	15
162	Pavlodar region	Communal State Enterprise on the right of economic management "Hospice of Pavlodar region" Health Department of Pavlodar region, Akimat of Pavlodar region	Palliative care	Other income	16
163	Pavlodar region	Communal State Enterprise on the right of economic management "Hospice of Pavlodar region" Health Department of Pavlodar region, Akimat of Pavlodar region	Palliative care	Republican (BP 067, PP 100) "Transfers of the Federal Migration Service to pay for the state budget".	67
164	Pavlodar region	Communal State Enterprise on the right of economic management "Hospice of Pavlodar region" Health Department of Pavlodar region, Akimat of Pavlodar region	Palliative care	Republican (BP 067, PP 100) "Transfers of the Federal Migration Service to pay for the state budget".	10
165	North Kazakhstan region	Communal state enterprise on the right of economic management "Multidisciplinary Regional Hospital" communal state institution "Department of Health Akimat of the North Kazakhstan region".	Palliative care	Republican (BP 067, PP 100) Oncology	3
166	North Kazakhstan region	Communal state enterprise on the right of economic management "Multidisciplinary Regional Hospital" communal state institution "Department of Health Akimat of the North Kazakhstan region".	Palliative care	Republican (BP 067, PP 100) Oncology	2
167	North Kazakhstan region	Communal state enterprise on the right of economic management "Multidisciplinary Regional Hospital" communal state institution "Department of Health Akimat of the North Kazakhstan region".	Palliative care	Republican (BP 067, PP 100) Oncology	1
168	North Kazakhstan region	Communal state enterprise on the right of economic management "Multidisciplinary Regional Hospital" communal state institution "Department of Health Akimat of the North Kazakhstan region".	Palliative care	Republican (BP 067, PP 100) "Transfers of the Federal Migration Service to pay for the state budget".	1
169	North Kazakhstan region	Communal state enterprise on the right of economic management "Multidisciplinary children's regional hospital" communal state institution "Department of Health Akimat of the North-Kazakhstan region".	Palliative care	Republican (BP 067, PP 100) "Transfers of the Federal Migration Service to pay for the state budget".	2
170	North Kazakhstan region	Communal state enterprise on the right of economic management "Multidisciplinary children's regional hospital" communal state institution "Department of Health Akimat of the North-Kazakhstan region".	Palliative care	Republican (BP 067, PP 100) "Transfers of the Federal Migration Service to pay for the state budget".	2
171	North Kazakhstan region	Communal state enterprise on the right of economic management "Ayirtau district hospital" communal state institution "Department of Health Akimat of the North Kazakhstan region	Palliative care	Republican (BP 067, PP 100) "Transfers of the Federal Migration Service to pay for the state budget".	2
172	North Kazakhstan region	Communal state enterprise on the right of economic management "Akzhar district hospital" communal state institution "Department of Health Akimat of the North Kazakhstan region".	Palliative care	Republican (BP 067, PP 100) "Transfers of the Federal Migration Service to pay for the state budget".	1
173	North Kazakhstan region	Communal state enterprise on the right of economic management "Akkaiyn district hospital" communal state institution "Department of Health Akimat of the North Kazakhstan region".	Palliative care	Republican (BP 067, PP 100) "Transfers of the Federal Migration Service to pay for the state budget".	1
174	North Kazakhstan region	Communal state enterprise on the right of economic management "Yavlenskaya district hospital" communal state institution "Department of Health Akimat of the North Kazakhstan region".	Palliative care	Republican (BP 067, PP 100) "Transfers of the Federal Migration Service to pay for the state budget".	1
175	North Kazakhstan region	Communal state enterprise on the right of economic management "Zhambyl district hospital" communal state institution "Department of Health Akimat of the North Kazakhstan region".	Palliative care	Republican (BP 067, PP 100) "Transfers of the Federal Migration Service to pay for the state budget".	2
176	North Kazakhstan region	Communal state enterprise on the right of economic management "District Hospital of Magzhan Zhumabaev district" communal state institution "Department of Health Akimat of the North Kazakhstan region".	Palliative care	Republican (BP 067, PP 100) "Transfers of the Federal Migration Service to pay for the state budget".	1
177	North Kazakhstan region	Communal state enterprise on the right of economic management "Kyzylzhar district hospital" communal state institution "Department of Health Akimat of the North Kazakhstan region	Palliative care	Republican (BP 067, PP 100) "Transfers of the Federal Migration Service to pay for the state budget".	1
178	North Kazakhstan region	Mamlyut district hospital" communal state enterprise on the right of economic management of the communal state institution "Department of Health of the Akimat of the North Kazakhstan region".	Palliative care	Republican (BP 067, PP 100) "Transfers of the Federal Migration Service to pay for the state budget".	1
179	North Kazakhstan region	Mamlyut district hospital" communal state enterprise on the right of economic management of the communal state institution "Department of Health of the Akimat of the North Kazakhstan region".	Palliative care	Republican (BP 067, PP 100) "Transfers of the Federal Migration Service to pay for the state budget".	1
180	North Kazakhstan region	Communal State Enterprise on the right of economic management "District Hospital of Gabit Musrepov district" communal state institution "Department of Health Akimat of the North Kazakhstan region".	Palliative care	Republican (BP 067, PP 100) "Transfers of the Federal Migration Service to pay for the state budget".	4
181	North Kazakhstan region	Communal state enterprise on the right of economic management "Tayynsha multi-district interdistrict hospital" communal state institution "Department of Health Akimat of the North Kazakhstan region".	Palliative care	Republican (BP 067, PP 100) "Transfers of the Federal Migration Service to pay for the state budget".	2
182	North Kazakhstan region	Communal state enterprise on the right of economic management "Timiryazevskaya district hospital" communal state institution "Department of Health Akimat of the North Kazakhstan region".	Palliative care	Republican (BP 067, PP 100) "Transfers of the Federal Migration Service to pay for the state budget".	1
183	North Kazakhstan region	Communal state enterprise on the right of economic management "Ualikhanovsky district hospital" communal state institution "Department of Health Akimat of the North Kazakhstan region".	Palliative care	Republican (BP 067, PP 100) "Transfers of the Federal Migration Service to pay for the state budget".	1
184	North Kazakhstan region	Communal state enterprise on the right of economic management "Ualikhanovsky district hospital" communal state institution "Department of Health Akimat of the North Kazakhstan region".	Palliative care	Republican (BP 067, PP 100) "Transfers of the Federal Migration Service to pay for the state budget".	1
185	North Kazakhstan region	Communal state enterprise on the right of economic management "District Hospital of Shal Akyn district" communal state institution "Department of Health Akimat of the North Kazakhstan region".	Palliative care	Republican (BP 067, PP 100) "Transfers of the Federal Migration Service to pay for the state budget".	2
186	North Kazakhstan region	Communal state enterprise on the right of economic management "District Hospital of Shal Akyn district" communal state institution "Department of Health Akimat of the North Kazakhstan region".	Palliative care	Republican (BP 067, PP 100) "Transfers of the Federal Migration Service to pay for the state budget".	1
187	North Kazakhstan region	Palliative Care Center Limited Liability Partnership	Palliative care	Republican (BP 067, PP 100) "Transfers of the Federal Migration Service to pay for the state budget".	35
188	East Kazakhstan region	Communal State Enterprise on the right of economic management "Kurchatov City Hospital" Health Department of East Kazakhstan region	Palliative care	Republican (BP 067, PP 100) "Transfers of the Federal Migration Service to pay for the state budget".	10

D
OC
24
ID
KZ
SI
U9
42
02
21
00
09
61
87
CD
E9
EF

189	East Kazakhstan region	Communal State Enterprise on the right of economic management "District Hospital № 2 of Kokpekty district" Health Department of East Kazakhstan region	Palliative care	Republican (BP 067, PP 100) village	1
190	East Kazakhstan region	Communal State Enterprise on the right of economic management "District Hospital of Kokpekty district" Health Department of East Kazakhstan region	Palliative care	Assets of the Fund on OSMS	1
191	East Kazakhstan region	Communal State Enterprise on the right of economic management "District Hospital of Kokpekty district" Health Department of East Kazakhstan region	Palliative care	Assets of the Fund on OSMS	1
192	East Kazakhstan region	Communal state enterprise on the right of economic management East Kazakhstan regional multidisciplinary "Center of Oncology and Surgery" of the Department of Health of the East Kazakhstan region	Palliative care	Paid services	2
193	East Kazakhstan region	Communal state enterprise on the right of economic management East Kazakhstan regional multidisciplinary "Center of Oncology and Surgery" of the Department of Health of the East Kazakhstan region	Palliative care	Republican (BP 067, PP 100) Oncology	8
194	East Kazakhstan region	Communal state enterprise on the right of economic management East Kazakhstan regional multidisciplinary "Center of Oncology and Surgery" of the Department of Health of the East Kazakhstan region	Palliative care	Republican (BP 067, PP 100) "Transfers of the Federal Migration Service to pay for the state budget".	2
195	East Kazakhstan region	Communal State Enterprise on the right of economic management "Center for Nuclear Medicine and Oncology of Semey City" of the Health Department of East Kazakhstan region	Palliative care	Republican (BP 067, PP 100) "Transfers of the Federal Migration Service to pay for the state budget".	7
196	East Kazakhstan region	Institution "Hospital of Palliative Care and Nursing Care of the Red Crescent Society of the Republic of Kazakhstan	Palliative care	Republican (BP 067, PP 100) "Transfers of the Federal Migration Service to pay for the state budget".	55
197	East Kazakhstan region	Institution "East Kazakhstan Regional Hospice	Palliative care	Republican (BP 067, PP 100) "Transfers of the Federal Migration Service to pay for the state budget".	24
198	East Kazakhstan region	Communal state enterprise on the right of economic management "City hospital № 4 of Ust-Kamenogorsk". Management of public health services of the East Kazakhstan region	Palliative care	Republican (BP 067, PP 100) "Transfers of the Federal Migration Service to pay for the state budget".	2
199	East Kazakhstan region	Semey City Hospice Communal State Enterprise of the Health Department of the East Kazakhstan Region	Palliative care	Republican (BP 067, PP 100) "Transfers of the Federal Migration Service to pay for the state budget".	1
200	East Kazakhstan region	Semey City Hospice Communal State Enterprise of the Health Department of the East Kazakhstan Region	Palliative care	Republican (BP 067, PP 100) "Transfers of the Federal Migration Service to pay for the state budget".	29
201	East Kazakhstan region	Semey Medical University, a non-profit joint-stock company	Palliative care	Republican (BP 067, PP 100) "Transfers of the Federal Migration Service to pay for the state budget".	1
202	Almaty g.a.	Communal State Enterprise on the right of economic management "City Center for Palliative Care" of the Department of Public Health of the city of Almaty	Palliative care	Paid services	25
203	Almaty g.a.	Communal State Enterprise on the right of economic management "City Center for Palliative Care" of the Department of Public Health of the city of Almaty	Palliative care	Republican (BP 067, PP 100) "Transfers of the Federal Migration Service to pay for the state budget".	60
204	Almaty g.a.	Communal State Enterprise on the right of economic management "City Center for Palliative Care" of the Department of Public Health of the city of Almaty	Palliative care	Republican (BP 067, PP 100) "Transfers of the Federal Migration Service to pay for the state budget".	20
205	Almaty g.a.	Communal State Enterprise on the right of economic management "City Center for Palliative Care" of the Department of Public Health of the city of Almaty	Palliative care	Republican (BP 067, PP 100) "Transfers of the Federal Migration Service to pay for the state budget".	30
206	Almaty g.a.	Communal State Enterprise on the right of economic management "City Center for Palliative Care" of the Department of Public Health of the city of Almaty	Palliative care	Republican (BP 067, PP 100) "Transfers of the Federal Migration Service to pay for the state budget".	30
207	Turkestan region	Turkestan City Central Hospital State Communal Enterprise on the right of economic management of the Turkestan Oblast Public Health Department	Palliative care	Republican (BP 067, PP 100) "Transfers of the Federal Migration Service to pay for the state budget".	1
208	Turkestan region	State communal enterprise on the right of economic management "Turkestan city central hospital" Public Health Department of Turkestan region	Palliative care	Republican (BP 067, PP 100) "Transfers of the Federal Migration Service to pay for the state budget".	1
209	Turkestan region	Turkestan City Central Hospital State Communal Enterprise on the right of economic management of the Turkestan Oblast Public Health Department	Palliative care	Republican (BP 067, PP 100) "Transfers of the Federal Migration Service to pay for the state budget".	2
210	Turkestan region	Institution "Clinical and Diagnostic Center of the International Kazakh-Turkish University named after Khoja Ahmet Yasawi	Palliative care	Republican (BP 067, PP 100) "Transfers of the Federal Migration Service to pay for the state budget".	3
211	Turkestan region	Institution "Clinical and Diagnostic Center of the International Kazakh-Turkish University named after Khoja Ahmet Yasawi	Palliative care	Republican (BP 067, PP 100) "Transfers of the Federal Migration Service to pay for the state budget".	2
212	Turkestan region	State communal enterprise on the right of economic management "Kentau City Central Hospital" of the Department of Public Health of Turkestan region	Palliative care	Republican (BP 067, PP 100) "Transfers of the Federal Migration Service to pay for the state budget".	5
213	Turkestan region	State communal enterprise on the right of economic management "Arys Central District Hospital" of the Department of Public Health of Turkestan region	Palliative care	Republican (BP 067, PP 100) "Transfers of the Federal Migration Service to pay for the state budget".	1
214	Turkestan region	State communal enterprise on the right of economic management "Arys Central District Hospital" of the Department of Public Health of Turkestan region	Palliative care	Republican (BP 067, PP 100) "Transfers of the Federal Migration Service to pay for the state budget".	1

D
OC
24
ID
KZ
SI
U9
42
02
21
00
09
61
87
CD
E9
EF

215	Turkestan region	State communal enterprise on the right of economic management "Kazygurt Central District Hospital" Public Health Department of Turkestan region	Palliative care	Republican (BP 067, PP 100) "Transfers of the Federal Migration Service to pay for the state budget".	1
216	Turkestan region	State communal enterprise on the right of economic management "Kazygurt Central District Hospital" Public Health Department of Turkestan region	Palliative care	Republican (BP 067, PP 100) "Transfers of the Federal Migration Service to pay for the state budget".	1
217	Turkestan region	State communal enterprise on the right of economic management "Baidibek Central District Hospital" of the Public Health Department of Turkestan Region	Palliative care	Republican (BP 067, PP 100) "Transfers of the Federal Migration Service to pay for the state budget".	1
218	Turkestan region	State communal enterprise on the right of economic management "Baidibek Central District Hospital" of the Public Health Department of Turkestan Region	Palliative care	Republican (BP 067, PP 100) "Transfers of the Federal Migration Service to pay for the state budget".	1
219	Turkestan region	State communal enterprise on the right of economic management "Zhetysay Central District Hospital" Public Health Department of Turkestan region	Palliative care	Republican (BP 067, PP 100) "Transfers of the Federal Migration Service to pay for the state budget".	3
220	Turkestan region	State communal enterprise on the right of economic management "Zhetysay district hospital "Asyk-Ata" of the Department of Public Health of Turkestan region	Palliative care	Republican (BP 067, PP 100) "Transfers of the Federal Migration Service to pay for the state budget".	2
221	Turkestan region	Maktaaral District Hospital "Myrzaakent" State Communal Enterprise on the right of economic management of the Public Health Department of Turkestan Region	Palliative care	Republican (BP 067, PP 100) "Transfers of the Federal Migration Service to pay for the state budget".	2
222	Turkestan region	State communal enterprise on the right of economic management "Maktaaral district hospital "Atakent" of the Department of Public Health of Turkestan region	Palliative care	Republican (BP 067, PP 100) village	2
223	Turkestan region	State communal enterprise on the right of economic management "Ordabasin central district hospital" of the public health department of Turkestan region	Palliative care	Republican (BP 067, PP 100) "Transfers of the Federal Migration Service to pay for the state budget".	3
224	Turkestan region	State communal enterprise on the right of economic management "Otrar Central District Hospital" Public Health Department of Turkestan region	Palliative care	Republican (BP 067, PP 100) "Transfers of the Federal Migration Service to pay for the state budget".	1
225	Turkestan region	State communal enterprise on the right of economic management "Otrar Central District Hospital" Public Health Department of Turkestan region	Palliative care	Republican (BP 067, PP 100) "Transfers of the Federal Migration Service to pay for the state budget".	1
226	Turkestan region	State communal enterprise on the right of economic management "Sairam Central District Hospital" of the Public Health Department of Turkestan Region	Palliative care	Republican (BP 067, PP 100) "Transfers of the Federal Migration Service to pay for the state budget".	1
227	Turkestan region	State communal enterprise on the right of economic management "Sairam Central District Hospital" of the Public Health Department of Turkestan Region	Palliative care	Republican (BP 067, PP 100) "Transfers of the Federal Migration Service to pay for the state budget".	1
228	Turkestan region	State communal enterprise on the right of economic management "Sairam Central District Hospital" of the Public Health Department of Turkestan Region	Palliative care	Republican (BP 067, PP 100) "Transfers of the Federal Migration Service to pay for the state budget".	1
229	Turkestan region	State communal enterprise on the right of economic management "Sairam district hospital "Karabulak" of the Department of Public Health of Turkestan region	Palliative care	Republican (BP 067, PP 100) village	1
230	Turkestan region	State communal enterprise on the right of economic management "Sairam district hospital "Karabulak" of the Department of Public Health of Turkestan region	Palliative care	Republican (BP 067, PP 100) village	1
231	Turkestan region	State communal enterprise on the right of economic management "Sairam district hospital "Karabulak" of the Department of Public Health of Turkestan region	Palliative care	Republican (BP 067, PP 100) village	1
232	Turkestan region	State communal enterprise on the right of economic management "Sairam district hospital "Karabulak" of the Department of Public Health of Turkestan region	Palliative care	Republican (BP 067, PP 100) "Transfers of the Federal Migration Service to pay for the state budget".	1
233	Turkestan region	Tolebi District Hospital, a state communal enterprise under the right of economic management of the Public Health Department of Turkestan Oblast	Palliative care	Republican (BP 067, PP 100) "Transfers of the Federal Migration Service to pay for the state budget".	1
234	Turkestan region	Tolebi District Hospital, a state communal enterprise under the right of economic management of the Public Health Department of Turkestan Oblast	Palliative care	Republican (BP 067, PP 100) "Transfers of the Federal Migration Service to pay for the state budget".	5
235	Turkestan region	State communal enterprise on the right of economic management "Tulkubas Central District Hospital" Public Health Department of Turkestan region	Palliative care	Republican (BP 067, PP 100) "Transfers of the Federal Migration Service to pay for the state budget".	2
236	Turkestan region	State communal enterprise on the right of economic management "Shardara Central District Hospital" Public Health Department of Turkestan region	Palliative care	Republican (BP 067, PP 100) "Transfers of the Federal Migration Service to pay for the state budget".	1
237	Turkestan region	State communal enterprise on the right of economic management "Shardara Central District Hospital" Public Health Department of Turkestan region	Palliative care	Republican (BP 067, PP 100) "Transfers of the Federal Migration Service to pay for the state budget".	1

D
OC
24
ID
KZ
SI
U9
42
02
21
00
09
61
87
CD
E9
EF

238	Turkestan region	Turkestan City Children's Hospital, a state-owned communal enterprise under the right of economic management of the Turkestan Oblast Public Health Department	Palliative care	Republican (BP 067, PP 100) "Transfers of the Federal Migration Service to pay for the state budget".	4
239	r. Shymkent	State communal enterprise on the right of economic management "City Children's Clinical Hospital" UZ Shymkent	Palliative care	Republican (BP 067, PP 100) "Transfers of the Federal Migration Service to pay for the state budget".	15
240	r. Shymkent	State communal enterprise on the right of economic management "Hyperbaric oxygenation center named after T.O. Orynbayev" UZ Shymkent	Palliative care	Republican (BP 067, PP 100) "Transfers of the Federal Migration Service to pay for the state budget".	45
241	r. Shymkent	State communal enterprise on the right of economic management "Municipal Cancer Center" UZ Shymkent	Palliative care	Republican (BP 067, PP 100) "Transfers of the Federal Migration Service to pay for the state budget".	23
242	Nur-Sultan	State communal enterprise on the right of economic management "Multidisciplinary City Children's Hospital №1" Nur-Sultan Akimat	Palliative care	Republican (BP 067, PP 100) "Transfers of the Federal Migration Service to pay for the state budget".	1
243	Nur-Sultan	State communal enterprise on the right of economic management "Multidisciplinary City Children's Hospital №1" Nur-Sultan Akimat	Palliative care	Republican (BP 067, PP 100) "Transfers of the Federal Migration Service to pay for the state budget".	1
244	Nur-Sultan	State communal enterprise on the right of economic management "Multidisciplinary City Children's Hospital No. 2" Nur-Sultan Akimat	Palliative care	Republican (BP 067, PP 100) "Transfers of the Federal Migration Service to pay for the state budget".	6
245	Nur-Sultan	State communal enterprise on the right of economic management "Multidisciplinary Medical Center" Nur-Sultan City Akimat	Palliative care	Republican (BP 067, PP 100) Oncology	30
246	Nur-Sultan	Limited Liability Partnership "AMARA Investment	Palliative care	Paid services	10
247	Nur-Sultan	Limited Liability Partnership "AMARA Investment	Palliative care	Republican (BP 067, PP 100) "Transfers of the Federal Migration Service to pay for the state budget".	100
248	Nur-Sultan	Adal Niyet Astana Private Charitable Foundation	Palliative care	Republican (BP 067, PP 100) "Transfers of the Federal Migration Service to pay for the state budget".	5

2481925

D
OC
24
ID
KZ
SI
U9
42
02
21
00
09
61
87
CD
E9
EF

0
0
0
0
0
0
0
0
0
0
0
0
0
0
0
0
0
0
0
0

0

0

0

0

0

0

0

0

0

0

0

0

0

0

0

0

0

0

0

0

DOC24 ID KZSIU942022100096187CDE9EF

0
0
0
0
0
0
0
0
0
0
0
0
0
0
0
0
0
0
0
0

0

0

0

0

0

0

0

0

0

0

0

0

0

0

0

0

0

0

0

0

0
0
0
0
0
0
0
0
0
0
0
0
0
0
0
0
0
0
0
0

0

0

0

0

0

0

0

0

0

0

0

0

0

0

0

0

0

0

0

0

0
0
0
0
0
0
0
0
0
0
0
0
0
0
0
0
0
0
0
0

0

0

0

0

0

0

0

0

0

0

0

0

0

0

0

0

0

0

0

0

0
0
0
0
0
0
0
0
0
0
0
0
0
0
0
0
0
0
0
0

0
0
0
0
0
0
0
0
0
0
0
0
0
0
0
0
0
0
0
0

0
0
0
0
0
0
0
0
0
0
0
0
0
0
0
0
0
0
0
0

0
0
0
0
0
0
0
0
0
0
0
0
0
0
0
0
0
0
0
0

0
0
0
0
0
0
0
0
0
0
0
0
0
0
0
0
0
0
0
0

0
0
0
0
0
0
0
0
0
0
0
0
0
0
0
0
0
0
0
0

0
0
0
0
0
0
0
0
0
0
0
0
0
0
0
0
0
0
0
0

0
0
0
0
0
0
0
0
0
0
0
0
0
0
0
0
0
0
0
0

0
0
0
0
0
0
0
0
0
0
0
0
0
0
0
0
0
0
0
0

0
0
0
0
0
0
0
0
0
0
0
0
0
0
0
0
0
0
0
0

0
0
0
0
0
0
0
0
0
0
0
0
0
0
0
0
0
0
0
0

0
0
0
0
0
0
0
0
0
0
0
0
0
0
0
0
0
0
0
0

0
0
0
0
0
0
0
0
0
0
0
0
0
0
0
0
0
0
0
0

0
0
0
0
0
0
0
0
0
0
0
0
0
0
0
0
0
0
0
0

0
0
0
0
0
0
0
0
0
0
0
0
0
0
0
0
0
0
0
0

0
0
0
0
0
0
0
0
0
0
0
0
0
0
0
0
0
0
0
0

0
0
0
0
0
0
0
0
0
0
0
0
0
0
0
0
0
0
0
0

0
0
0
0
0
0
0
0
0
0
0
0
0
0
0
0
0
0
0
0

0
0
0
0
0
0
0
0
0
0
0
0
0
0
0
0
0
0
0
0

0

0

0

0

0

0

0

0

0

0

0

0

0

0

0

0

0

0

0

0

0
0
0
0
0
0
0
0
0
0
0
0
0
0
0
0
0
0
0
0

0
0
0
0
0
0
0
0
0
0
0
0
0
0
0
0
0
0
0
0

0
0
0
0
0
0
0
0
0
0
0
0
0
0
0
0
0
0
0
0

0
0
0
0
0
0
0
0
0
0
0
0
0
0
0
0
0
0
0
0

0
0
0
0
0
0
0
0
0
0
0
0
0
0
0
0
0
0
0
0

0
0
0
0
0
0
0
0
0
0
0
0
0
0
0
0
0
0
0
0

0
0
0
0
0
0
0
0
0
0
0
0
0
0
0
0
0
0
0
0

0
0
0
0
0
0
0
0
0
0
0
0
0
0
0
0
0
0
0
0

0
0
0
0
0
0
0
0
0
0
0
0
0
0
0
0
0
0
0
0

0
0
0
0
0
0
0
0
0
0
0
0
0
0
0
0
0
0
0
0

DOC24 ID KZSIU942022100096187CDE9EF

0
0
0
0
0
0
0
0
0
0
0
0
0
0
0
0
0
0
0
0

DOC24 ID KZSIU942022100096187CDE9EF

0

0

0

0

0

0

0

0

0

0

0

0

0

0

0

0

0

0

0

0

0
0
0
0
0
0
0
0
0
0
0
0
0
0
0
0
0
0
0
0

0
0
0
0
0
0
0
0
0
0
0
0
0
0
0
0
0
0
0
0

0
0
0
0
0
0
0
0
0
0
0
0
0
0
0
0
0
0
0
0

0
0
0
0
0
0
0
0
0
0
0
0
0
0
0
0
0
0
0
0

0
0
0
0
0
0
0
0
0
0
0
0
0
0
0
0
0
0
0
0

0
0
0
0
0
0
0
0
0
0
0
0
0
0
0
0
0
0
0
0

0
0
0
0
0
0
0
0
0
0
0
0
0
0
0
0
0
0
0
0

0
0
0
0
0
0
0
0
0
0
0
0
0
0
0
0
0
0
0
0

0

0

0

0

0

0

0

0

0

0

0

0

0

0

0

0

0

0

0

0

DOC24 ID KZSIU942022100096187CDE9EF

0
0
0
0
0
0
0
0
0
0
0
0
0
0
0
0
0

This electronic document DOC24 ID KZSIU942022100096187CDE9EF is signed with an electronic digital signature and sent through the information system "Kazakhstan Electronic Document Exchange Center" Doculite.kz.

To check the electronic document, go to:
https://doculite.kz/landing?verify=KZSIU942022100096187CDE9EF

**Document type**	Outgoing document
**Document number and date**	No. 315 of 14.03.2022.
**Organization/sender**	THE NATIONAL CENTER FOR HEALTH CARE DEVELOPMENT, RCHD MINISTRY OF HEALTH OF THE REPUBLIC OF KAZAKHSTAN".
**Recipient(s)**	KAZAKHSTAN PALLIATIVE CARE ASSOCIATION
**Electronic digital document signatures**	Agreed:<br>without EDS<br>Signing time: 11.03.2022 10:37<br><br>Agreed:<br>without EDS<br>Signing time: 11.03.2022 13:55<br><br>REPUBLICAN STATE ENTERPRISE ON THE RIGHT OF ECONOMIC MANAGEMENT "SALIDAT KAIRBEKOVA NATIONAL SCIENTIFIC CENTER FOR HEALTH DEVELOPMENT" OF THE MINISTRY OF HEALTH OF THE REPUBLIC OF KAZAKHSTAN<br>Signed: AITUAROVA DANA<br>MIIW1wYJ...7PIDygg==<br>Signing time: 11.03.2022 18:00<br><br>REPUBLICAN STATE ENTERPRISE ON THE RIGHT OF ECONOMIC MANAGEMENT "SALIDAT KAIRBEKOVA NATIONAL SCIENTIFIC CENTER FOR HEALTH DEVELOPMENT" OF THE MINISTRY OF HEALTH OF THE REPUBLIC OF KAZAKHSTAN<br>EDS of the Chancellery: Chief Specialist ABDUHALIKOVA NURJAMAL<br>MIIXEAYJ...7S04KCQ==<br>Signing time: 14.03.2022 09:25

This document under paragraph 1 of Article 7 of the Law of January 7, 2003 N 370-II "On Electronic Document and Digital Signature" certified by an electronic digital signature of the person authorized to sign it, is equivalent to a signed document on paper.

DOC24 ID KZSIU942022100096187CDE9EF
