# Supplementary material for: Challenges for Developing Palliative Care Services in Resource- Limited Settings of Kazakhstan
Source: Public Health Rev. 2023 Aug 18;44:1605672. doi: 10.3389/phrs.2023.1605672 (PMC10476099; doi:10.3389/phrs.2023.1605672)
Supplement: Supplementary file 3 [file Table2.pdf]

Number of patients registered for dispensary care in the context of diseases in the Republic of Kazakhstan according to the information system "Electronic register of patients for dispensary care" as of 01.01.2022.

№	Disease classes	Nosological forms	ICD 10* codes	Number of patients registered for medical check-ups	Total
**Total registered**					
1	Diseases of the endocrine system, eating disorders and metabolic disorders	Type 1 diabetes mellitus	E10	25798	417328
		Type 2 diabetes mellitus	E11	391530	
2	Diseases of the nervous system	Parkinson's disease	G20	9114	17559
		Alzheimer's disease	G30	494	
		Other degenerative diseases of the nervous system	G31	151	
		Unspecified encephalopathy	G93.4	7800	
3	Diseases of the circulatory system	Primary pulmonary hypertension	I27.0	1459	177182
		Heart failure	I50	138941	
		Consequences of cerebrovascular disease	I69	36782	
4	Respiratory diseases	Other chronic obstructive pulmonary disease	J44	87006	87006
5	Diseases of the digestive organs	Toxic liver damage with a pattern of other liver disorders	K71.7	75	6100
		Liver failure not classified under other headings	K72	103	
		Fibrosis and cirrhosis of the liver	K74	5922	
6	Diseases of the urogenital system	Chronic kidney disease	N18	11832	11832
7	Injuries	Injuries	S06, S30- S39	506	506
**Children (0 to 17 years old inclusive)**					
8	Diseases of the endocrine system, eating disorders and metabolic disorders	Type 1 diabetes mellitus	E10	4328	6564
		Type 2 diabetes mellitus	E11	294	
		Gaucher disease	E75.2	16	
		Mucopolysaccharidoses	E76.0 E76.1 E76.2	60	
		Cystic fibrosis	E84.8	61	
		Phenylketonuria	E70.0	158	
		Hyperfunction of the pituitary gland	E22	406	
		Hypofunction of the pituitary gland	E23.0, E23.2	1241	
9	Diseases of the nervous system	Consequences of inflammatory diseases of the central nervous system	G09	899	19637
		Disease of the motor neuron	G12.2	10	
		Other degenerative diseases of the CNS	G30- G32	47	
		Demyelinating CNS diseases	G35- G37	57	
		Duchenne muscular dystrophy	G71	480	
		Cerebral palsy	G80	18144	
10	Diseases of the circulatory system	Chronic rheumatic heart disease	I05- I09	254	1678
		Heart failure	I50	153	
		Cardiomyopathy	I42	885	
		Subarachnoid hemorrhage	I60	89	
		Intracerebral hemorrhage	I61	162	
		Other non-traumatic intracranial hemorrhage	I62	26	
		Brain Infarction	I63	84	
		Stroke not specified as a hemorrhage or infarction	I64	25	
12	Diseases of the digestive organs	Non-infectious enteritis	K50- 52	297	364
		Liver failure not classified under other headings	K72	5	
		Fibrosis and cirrhosis of the liver	K74	62	
13	Diseases of the musculoskeletal system and connective tissue	Systemic connective tissue lesions	M30- M36	478	478
14	Diseases of the urogenital system	Renal failure	N18- N19	354	354
15	Congenital anomalies,<br>Deformities and chromosomal abnormalities	Congenital abnormalities of the nervous system	Q02	530	28381
		Congenital anomalies of the circulatory system	Q20- Q25	27555	
		Congenital ichthyosis	Q80	296	

Note: The list of diagnoses according to the order of MH RK from October 23, 2020 № КР ДСМ-149/2020 "About approval of rules of organization of medical care to persons with chronic diseases, frequency and terms of observation, obligatory minimum and multiplicity of diagnostic tests".

D
OC
24
ID
KZ
SI
U9
42
02
21
00
09
61
87
CD
E9
EF

This electronic document DOC24 ID KZSIU942022100096187CDE9EF is signed with an electronic digital signature and sent through the information system "Kazakhstan Electronic Document Exchange Center" Doculite.kz.

To check the electronic document, go to: https://doculite.kz/landing?verify=KZSIU942022100096187CDE9EF

**Document type**	Outgoing document
**Document number and date**	No. 315 of 14.03.2022.
**Organization/sender**	RPE "REPUBLICAN CENTER FOR HEALTH CARE DEVELOPMENT" MINISTRY OF HEALTH OF THE REPUBLIC OF KAZAKHSTAN".
**Recipient(s)**	KAZAKHSTAN PALLIATIVE CARE ASSOCIATION

**Electronic digital document signatures**	Agreed:<br>without EDS<br>Signing time: 11.03.2022 10:37<br><br>Agreed:<br>without EDS<br>Signing time: 11.03.2022 13:55<br><br>REPUBLICAN STATE ENTERPRISE ON THE RIGHT OF ECONOMIC MANAGEMENT "SALIDAT KAIRBEKOVA NATIONAL SCIENTIFIC CENTER FOR HEALTH DEVELOPMENT" OF THE MINISTRY OF HEALTH OF THE REPUBLIC OF KAZAKHSTAN<br>Signed: AITUAROVA DANA<br>MIIW1wYJ...7PIDygg==<br>Signing time: 11.03.2022 18:00<br><br>REPUBLICAN STATE ENTERPRISE ON THE RIGHT OF ECONOMIC MANAGEMENT "SALIDAT KAIRBEKOVA NATIONAL SCIENTIFIC CENTER FOR HEALTH DEVELOPMENT" OF THE MINISTRY OF HEALTH OF THE REPUBLIC OF KAZAKHSTAN<br>EDS of the Chancellery: Chief Specialist ABDUHALIKOVA NURJAMAL<br>MIIXEAYJ...7S04KCQ==<br>Signing time: 14.03.2022 09:25

This document under paragraph 1 of Article 7 of the Law of January 7, 2003 N 370-II "On Electronic Document and Digital Signature" certified by an electronic digital signature of the person authorized to sign it, is equivalent to a signed document on paper.

D
OC
24
ID
KZ
SI
U9
42
02
21
00
09
61
87
CD
E9
EF
